# Structural and Reproductive Adaptations in the Endemic *Silene zawadskii* in Response to Alpine Environmental Stress

**DOI:** 10.3390/plants15132062

**Published:** 2026-07-02

**Authors:** Irina Neta Gostin, Irinel Eugen Popescu

**Affiliations:** Faculty of Biology, “Alexandru Ioan Cuza” University of Iași, Bdul Carol I, No. 11, 700506 Iasi, Romania; irinellus@yahoo.com

**Keywords:** anatomical, endemic, calcium oxalate, high-altitude ecosystems, flower, pollination

## Abstract

*Silene zawadskii* Herbich is an endemic species restricted to the South-Eastern Carpathians, being found in Romania and Ukraine. The species is rare and protected in the two countries. The root system is deep, to facilitate water absorption, the basal leaves, arranged in a rosette, are leathery and glabrous. The stem presents numerous multicellular, uniseriate non-glandular trichomes, only in the upper part; they become caducous at the base. The small size of the plant, correlated with the very narrow xylem vessels, represents a way of increasing the resistance to physiological drought and preventing embolism in drier periods. Another particular feature of this species is the presence of numerous large calcium oxalate crystals (druses), both in the stem and especially in the leaves. The flowers are adapted to entomophilous pollination, showing structural traits that facilitate interactions with alpine pollinators, particularly under conditions of reduced insect diversity at high altitudes. These anatomical traits reflect its ecological specialization, making it a valuable indicator for assessing edaphic–climatic niche specificity and the functional connectivity of high-altitude ecological networks.

## 1. Introduction

**Figure 1:**
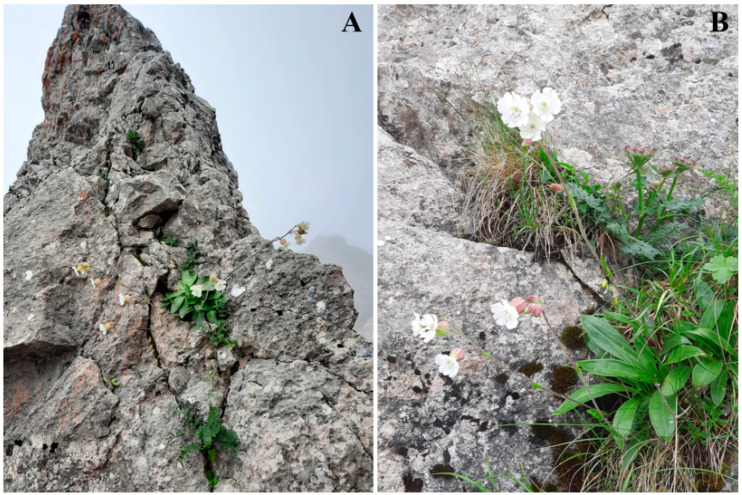
*Silene zawadskii* Herbich: (**A**) plant in natural habitat, “Rarău-Pietrele Doamnei” Nature Reserve, (**B**) close-up of the analyzed species.

The genus *Silene* belongs to the Caryophyllaceae family and is a large genus, with 890 accepted species, natively distributed on all continents, except Australia, where they were introduced [1,2]. *Silene zawadzkii* Herbich (Figure 1) is an endemic species in the South-Eastern Carpathians (in Romania and Ukraine) [1,2]. Populations of this species have also been reported in the Southern Carpathians, in Fagaras and Bucegi, which suggests that in the past its range was much wider [2].

The species has had four synonyms over time, being placed in the genera Melandrium, Elisanthe and Silenanthe; these synonyms are *Melandrium zawadskii* (Herbich) A.Braun, *Elisanthe zawadzkii* (Herbich) Fuss, (Isonym *Elisanthe zawadskii* (Herbich) Klokov), *Silenanthe zawadzkii* (Herbich) Griseb. and Schenk [2,3,4,5,6]. From a phylogenetic point of view, recent studies place *S. zawadskii* at the base of the *Physolychnis* clade, along with other diploid species that grow in the Arctic–Siberian zone (such as *S. ajanensis*, *S. linnaea*, *S. villosula*, *S. samojedorum*) [7]. However, most species in this clade are North American, polyploid, and genetically more distant from *S. zawadskii* [8].

Alpine areas are characterized by cold, harsh environments, climatic stress, reduced atmospheric pressure, reduced atmospheric temperature associated with the reduction in vapor pressure deficits, high solar radiation related to greater shortwaves [9,10,11].

The structural adaptations of plant species to living environments with special conditions, such as alpine and subalpine ones, have been an issue that has interested researchers over time [12,13,14,15]. Knowledge of the morpho-structural peculiarities of the species plays a vital role in understanding local biodiversity patterns and ecological specificity [14]. Plants that grow in harsh, limiting environments, such as the subalpine climate present specific, have variable, functional traits depending on the strategy adopted by each species. The key to survival under these conditions is finding a compromise between growth and the conservation of available resources [16]. Endemic plants have different distribution and ecology patterns compared to general biodiversity, which requires specific conservation strategies. Narrow niches and very specific habitat requirements make them particularly vulnerable to climate change [17].

Reproductive success is essential for plant survival in adverse environments. *S. zawadskii* reproduces predominantly sexually, through seeds [18] which have a high germination rate. Pollination of this species is entomophilous. Insect diversity of species and the abundance of the number of individuals decrease with the altitude and the activity is at low levels in the alpine areas [19]. Flower visitation rates decline with the increasing elevation [19,20,21,22]). Entomophilous cross-pollination in the alpine areas faces some difficulties in comparison with the same process in the lowerlands areas, like the short period of flowering, the activity of insects restricted to daytime and the limited pollinator availability [23,24].

All these environmental challenges must be solved by plants through specific adaptive strategies. This paper investigates in detail for the first time the structure and micromorphology of the endemic species *S. zawadzkii*, emphasizing morphogenetic aspects that lead to the construction of an integrated adaptive system, a functional biological “machinery” shaped to withstand unfavorable environmental conditions and secure species survival through multiple adaptive traits.

## 2. Results

### 2.1. Root Anatomy (Figure 2)

**Figure 2:**
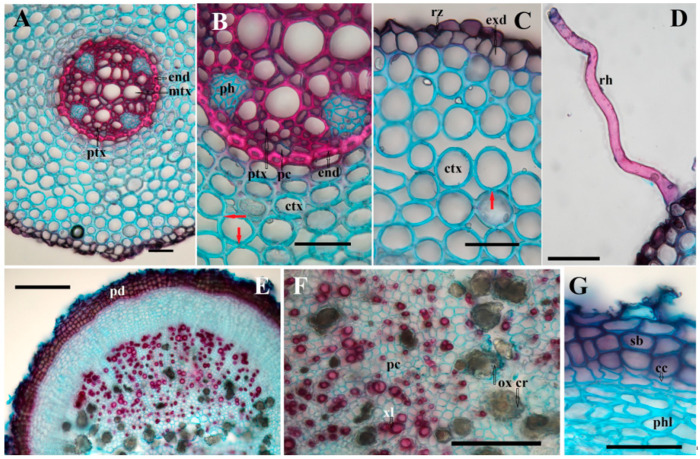
Cross-sections through the young root (**A**–**D**) and mature root (**E**–**G**): cc—cork cambium, ctx—cortex, end—endodermis, exd—exodermis, mtx—metaxylem, ox cr—oxalate crystal, pd—peridermis, ph—phloem, phl—phellodermis, ptx—protoxylem, rh—root hair, rz—rhizodermis, sb—suber, xl—xylem vessel, pc—placenta, the arrows indicate simple pits in the cell walls. Scale bar (**A**–**D**,**F**,**G**) = 50 µm, (**E**) = 100 µm.

The very thin roots already show the beginning of secondary growth. The central cylinder has a tetrarch structure. The xylem fascicles, four in number, are composed of 1–3 metaxylem vessels and a few protoxylem vessels towards the pericycle (Figure 2A,B).

The cortex is compact, formed by parenchymal cells with thickened primary walls and reduced air spaces between them (Figure 2C). The endoderm is predominantly of the secondary type at this level.

The rhizodermis still presents long, unbranched root hairs (Figure 2D). Suberization begins in the exodermis (Figure 2C).

The thicker roots are already protected by the periderm, formed following the activity of the cork cambium (Figure 2E). The suber is formed by 3–4 layers of tangentially elongated cells. The cork cambium is one- or two-layered, with 4–5 layers of slightly collenchymatous phelloderm located on the inner side (Figure 2G). The secondary xylem is well developed, with few wood vessels and a large amount of parenchyma (Figure 2F). Calcium oxalate druses are present in large quantities, both in the central part of the root and in the woody parenchyma cells—sometimes grouped in clusters of 3–5 crystals of variable size.

### 2.2. Stem Anatomy

**Figure 3:**
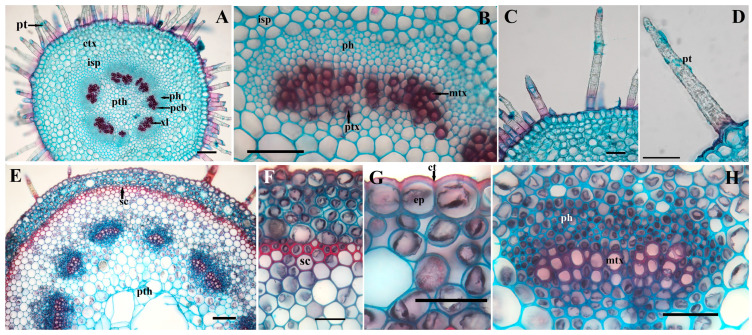
Cross-sections through the top of the stem, under the flower (**A**–**D**) and in the upper quarter (**E**–**H**): ct—cuticle, ctx—cortex, ep—epidermis, isp—initial sclerenchymatic pericycle, mtx—metaxilem, pcb—procambium, ph—phloem, pt—trichome, pth—pith, ptx—protoxylem, xl—xylem. Scale bar (**A**,**E**) = 100 µm, (**B**–**D**,**F**–**H**) = 50 µm.

**Figure 4:**
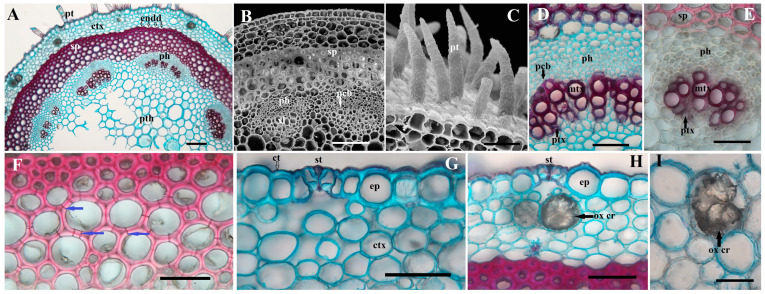
Cross-sections through the middle of the stem: (**A**,**D**,**G**–**I**) staining with Astra Blue and fucsine, (**B**,**C**) SEM micrographs, (**E**,**F**) staining with fluoroglucine and HCl, histochemical test for lignin. ct—cuticle, ctx—cortex, ep—epidermis, endd—endodermoid, mtx—metaxilem, ox cr—oxalate crystal, pcb—procambium, ph—phloem, pt—trichome, pth—pith, ptx—protoxylem, sp—sclerenchymatic pericycle, st—stomata, xl—xylem, the arrows indicate simple pits in the cell walls. Scale bar (**A**,**B**) = 100 µm, (**C**–**I**) = 50 µm.

**Figure 5:**
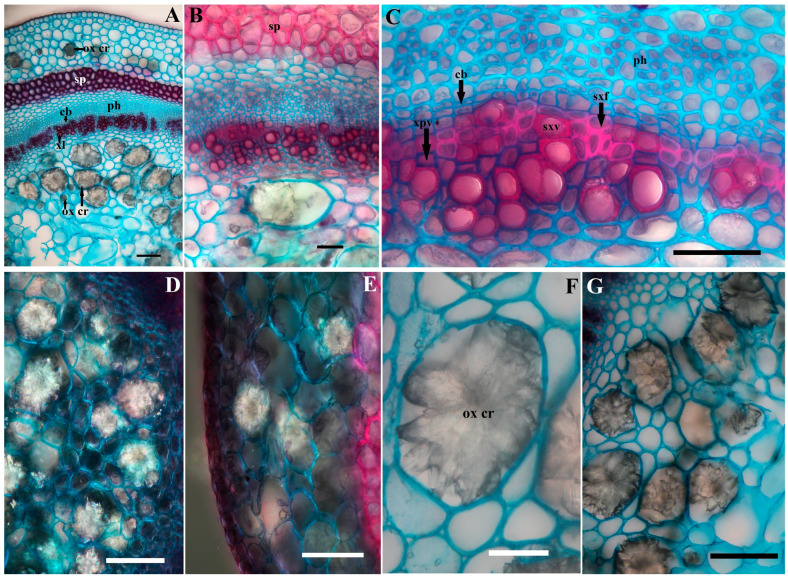
Cross-sections through the base of the stem ((**A**–**C**,**F**,**G**)—normal light, (**D**,**E**)—polarized light): cb—cambium, sxv—secondary xylem vessel, ox cr—oxalate crystal, ph—phloem, sp—sclerenchymatic pericycle, xl—xylem, xpv—xylem parenchyma cells, sxf—secondary xylem fibers. Scale bar (**A**) = 100 µm, (**B**–**G**) = 50 µm.

**Figure 6:**
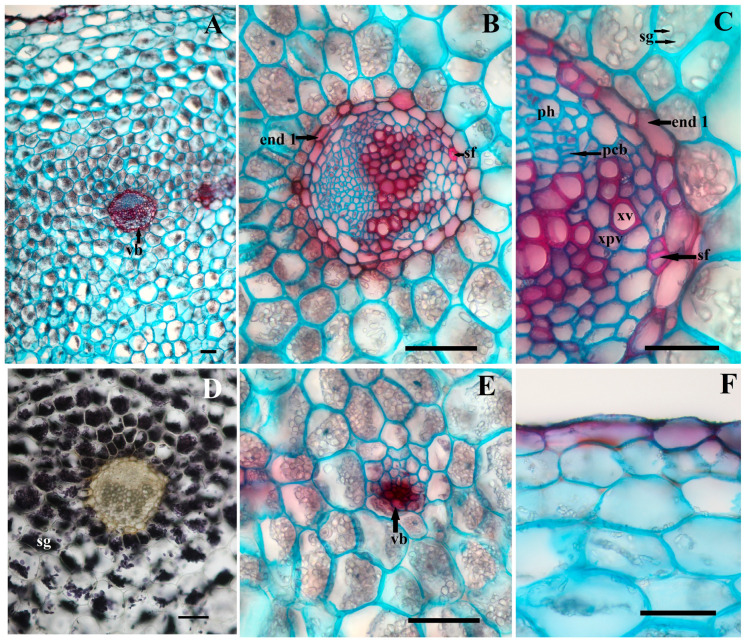
Cross-sections through the rhizome: overview (**A**), details of a vascular bundle (**B**,**C**), section stained with Lugol’s solution to highlight starch (**D**), small vascular bundle (**E**), external area of the rhizome (**F**). end 1—primary endodermis, pcb—procambium, ph—phloem, sg—starch grains, sf—sclerenchyma fibers, vb—vascular bundle, xv—xylem vessel, xpv—xylem parenchyma cells. Scale bar (**A**,**B**,**D**–**F**) = 100 µm, (**C**) = 50 µm.

Histo-anatomical analyses of the stem, carried out at different levels, from the tip to the base, have highlighted the complete edification of the primary structure and the beginning of the transition to the secondary structure (Figure 3, Figure 4, Figure 5 and Figure 6, Table 1).

The top of the stem has a circular shape in the cross-section (Figure 3A–D). The epidermis presents numerous multicellular, uniseriate non-glandular trichomes, formed by 3–6 elongated cells, the last one having a sharp tip. The surface of the trichomes is echinate, covered with micropapillae (Figure 3D).

The epidermis has approximately isodiametric cells in cross-section, with slightly thickened external walls and covered by a thin cuticle. The cortex is compact, formed by 5–7 layers of cells with decreasing diameter from the outside to the inside. In the central cylinder, the first layers of cells that will differentiate into a sclerenchymatous pericycle are compact, isodiametric with no intercellular spaces between them.

At this level the central cylinder is not separated by special structures from the cortex. Vascular bundles are 8–9 in number, arranged in a circle; these have variable sizes depending on their position relative to the base of the cauline leaves. The primary xylem is composed of a few protoxylem vessels, already collapsed due to the rapid elongation of the floral stem (Figure 3B), and 3–4 radial rows of metaxylem vessels with small diameters and strongly thickened, lignified walls. In fact, at this level the only lignified structures are represented by the xylem vessels. The pith is compact.

In the area between the middle and the top of the stem (Figure 3E–H), the lignification of the cells in the sclerenchymatous pericycle begins; the first 2–3 layers of cells in the central cylinder have thickened and lignified walls, while the remaining cells retain their cellulose–pectic walls and the characteristic shape observed at the stem apex (Figure 3F). The non-glandular trichomes become rare, and the external walls of the epidermal cells are slightly thickened, also covered by a thin cuticle. Vascular bundles contain primary xylem and primary phloem (Figure 3H).

In the middle area of the stem (Figure 4), structural changes occur that involve all anatomical areas. The external walls of the epidermal cells become thicker, as does the cuticle that covers them, in parallel with a reduction in the density of the non-glandular trichomes (Figure 4A,G). The cortex appears formed by a smaller number of cell layers 4–6 (Figure 4A,B).

A multi-layered sclerenchymatous pericycle appears, consisting of 6–7 layers of cells, with variable diameter, surrounding the vascular bundles (Figure 4A,F). These retain a primary structure, similar to that observed at the tip of the stem (Figure 4D,E). The pith appears slightly disorganized. Large compound crystals (druses) of calcium oxalate are visible in both the cortex and the pith (Figure 4H,I).

At the base of the stem (Figure 5), the transition to the secondary structure begins, at the level of the vascular tissues. The epidermis continues to provide protection for the stem (Figure 5A). The cortex and pith contain numerous calcium oxalate—druses, very large in size, which sometimes destroy the cells that formed them (Figure 5B,F,G). They appear in idioblasts, which are larger in size than the parenchymal cells in the cortex and pith. The crystals appear birefringent in polarized light (Figure 5D,E).

The cambium is well individualized, with intense activity, formed by 2–3 layers of flattened cells. Xylem sclerenchyma fibers (libriform) are visible in the secondary xylem, with moderately thickened and lignified walls. The secondary phloem elements have thickened, cellulose–pectic walls (Figure 5B,C). The pith cells in the vicinity of the primary xylem also have thickened primary walls (Figure 5C).

The rhizome has a circular outline in the cross-section (Figure 6). The epidermis consists of tangentially elongated cells, with thin external walls (Figure 6F). The ground parenchyma is compact, well developed, and formed by isodiametric cells, with small air spaces. The vascular bundles are formed by primary tissues. Each bundle is surrounded by a well-marked endoderm, of primary type (Figure 6B,C). Near the endodermis, in the internal part, isolated sclerenchyma fibers are observed from place to place (Figure 6C). The primary xylem consists of metaxylem vessels, with lignified walls, and protoxylem vessels that are already collapsed at this level. The procambium is well individualized. The phloem is formed by sieve tubes and companion cells with slightly thickened walls. Large quantities of starch granules are observed in all ground parenchyma, but are denser in the proximity of the vascular bundles; the starch grains show a positive reaction to Lugol’s solution (Figure 6D). Rare small vascular bundles are also observed in the cortex; they consist of a few conducting elements and lack an endodermis (Figure 6E).

### 2.3. Leaf Anatomy

Leaf morphogenesis was analyzed starting from the leaf primordia to the mature basal leaf. Also, the structure of the cauline leaves was investigated.

**Figure 7:**
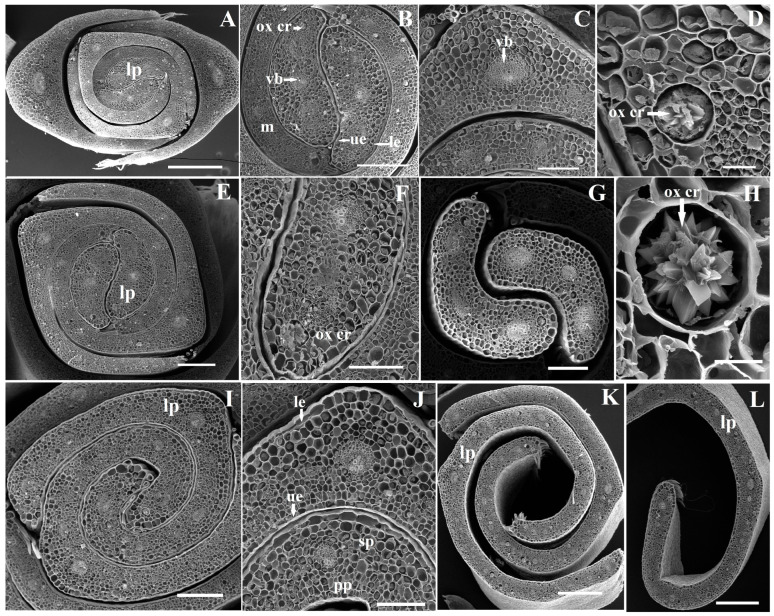
Cross-sections through leaf primordia (SEM micrographs): leaf primordia of 5—600 µm (**A**–**F**), leaf primordia of 1000—1100 µm (**G**,**H**), leaf primordia of 2100—2400 µm (**I**,**J**), leaf primordia over 5000 µm (**K**,**L**): ox cr—oxalate crystal, le—lower epidermis, lp—leaf primordia, m—mesophyll, sp—spongy parenchyma, pp—palisade parenchyma, ue—upper epidermis, vb—vascular bundle. Scale bar: (**A**,**K**,**L**) = 500 µm, (**B**,**E**,**G**,**I**) = 200 µm, (**C**,**F**,**J**) = 100 µm, (**D**,**H**) = 20 µm.

Leaf primordia of 5–600 µm have three vascular bundles in development (Figure 7A–F). At the level of the midrib bundle, a few xylem vessels are observed (with a 7–9 µm diameter); otherwise, it has not differentiated. The mesophyll is compact, with cells still undifferentiated; the number of cell layers is 8–10. However, in the marginal areas of the leaf, large calcium oxalate druses are already observed (with an average diameter of 20–30 µm).

In 1000–1100 µm primordia, differentiation of the palisade tissue beneath the upper epidermis begins (Figure 7G,H). In the three vascular bundles, xylem and phloem with a primary structure are observed. The xylem vessel diameters vary from 12 µm to 19 µm. The calcium oxalate crystals have an average diameter of 45 µm.

In 2100–2400 µm primordia (Figure 7I,J) the number of vascular bundles increases to 10–12, and the of non-glandular trichomes begins in the marginal regions of the leaf blade. The expansion of the leaf blade leads to the spiral positioning of the primordia relative to each other.

In the 5000 µm leaf primordia (Figure 7K,L), the leaf structure is almost completely formed. Vascular bundles are numerous, of different sizes corresponding to the veins of different orders. The assimilatory tissue is differentiated into palisade tissue (single or bi-layered) under the upper epidermis (cells with a height between 25 and 50 µm) and spongy parenchyma formed by 7–8 layers of rounded cells. Calcium oxalate crystals are numerous, with dimensions of 35–45 µm.

**Figure 8:**
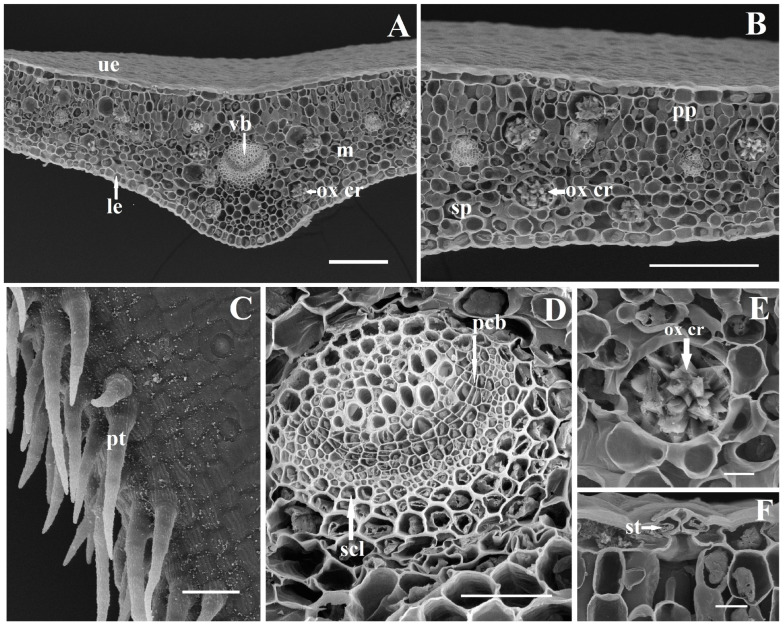
Cross-sections through mature basal leaf (SEM micrographs): overall view near the midrib (**A**), detail between the ribs (**B**), leaf margin (**C**), vascular bundle (**D**), calcium oxalate crystal (**E**), stomata in the upper epidermis (**F**): ox cr—oxalate crystal, le—lower epidermis, m—mesophyll, sp—spongy parenchyma, pcb—procambiun, pp—palisade parenchyma, pt—trichome, scl—sclerenchyma, st—stomata, ue—upper epidermis, vb—vacular bundle. Scale bar: (**A**,**B**) = 200 µm, (**C**) = 100 µm, (**D**) = 50 µm, (**E**,**F**) = 20 µm.

**Figure 9:**
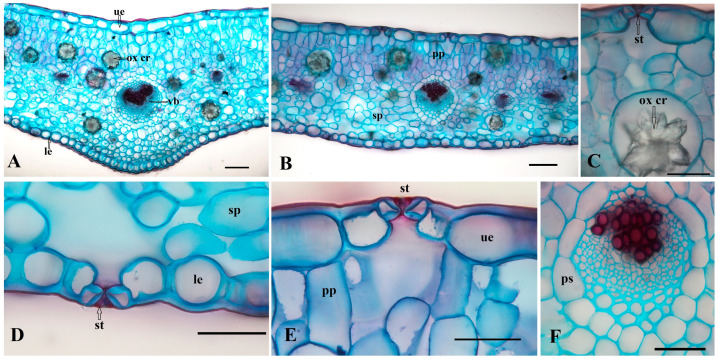
Cross-sections through mature basal leaf: midrib (**A**), lateral vein (**B**), calcium oxalate crystal in mesophyll (**C**), stomata in the lower epidermis (**D**), stomata in the upper epidermis (**E**), detail of vascular bundle in the secondary vein (**F**): ox cr—oxalate crystal, le—lower epidermis, sp—spongy parenchyma, st—stomata, ps—parenchymatous sheath, pp—palisade parenchyma, ue—upper epidermis, vb—vascular bundle. Scale bar: (**A**,**B**) = 100 µm, (**C**–**F**) = 500 µm.

**Figure 10:**
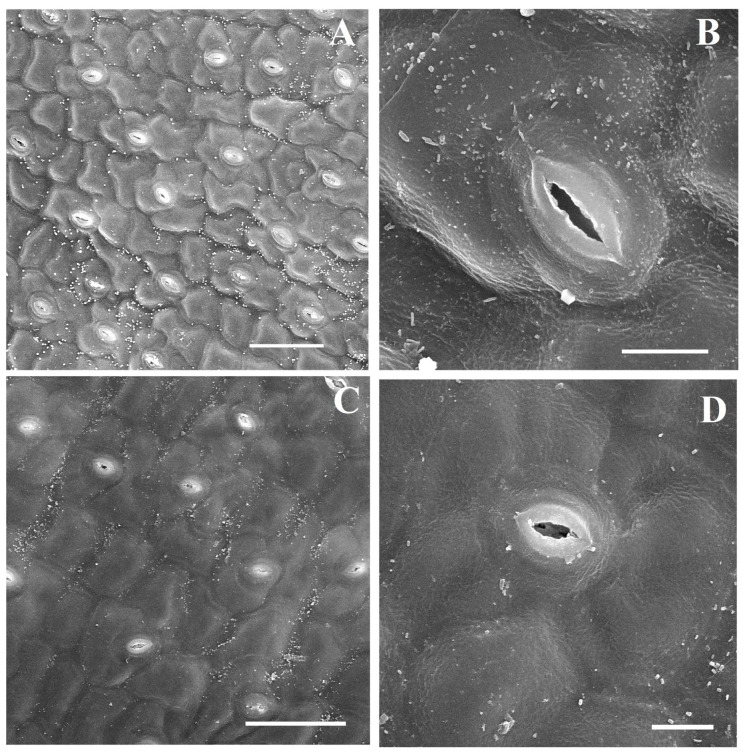
Epidermises (SEM micrographs): (**A**,**B**)—upper epidermis, (**C**,**D**)—lower epidermis. Scale bar: (**A**,**C**) = 100 µm, (**B**,**D**) = 20 µm.

Mature basal leaves have a bifacial structure (Figure 8, Figure 9 and Figure 10). Xylem vessels have a diameter of 12–20 µm. Calcium oxalate crystals have diameters between 50 and 100 µm. Palisade cells do not exceed 50 µm, remaining short even in adult leaves.

Vascular bundles both in the midrib and in the higher order veins are surrounded by a parenchymatous sheath formed by ordered isodiametric cells (Figure 9B,F). Stomata are present in both epidermises, being more frequent in the lower epidermis (Figure 8D,E and Figure 10B,D).

**Figure 11:**
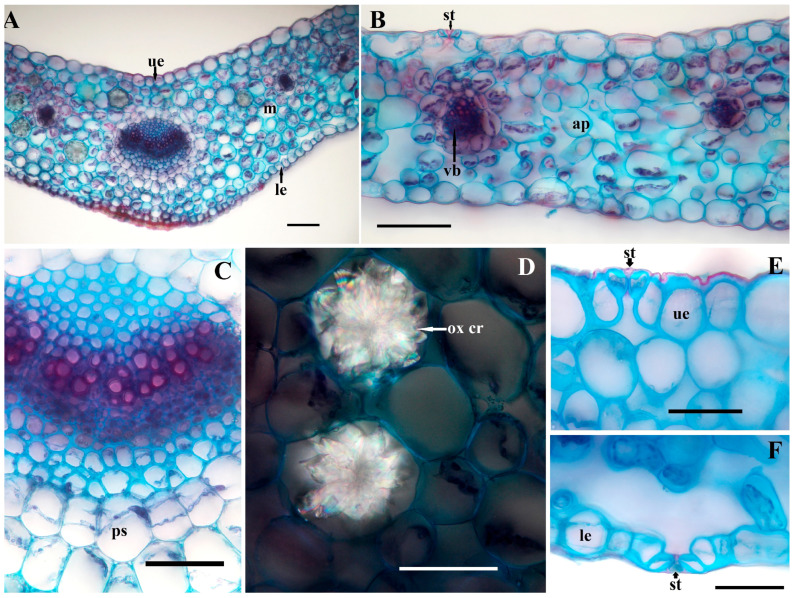
Cross-sections through cauline mature leaf ((**A**–**C**,**E**,**F**) normal light, (**D**) polarized light): ap—assimilatory parenchyma, ox cr—oxalate crystal, le—lower epidermis, m—mesophyll, st—stomata, ps—parenchymatous sheath, ue—upper epidermis, vb—vascular bundle. Scale bar: (**A**,**B**) = 100 µm, (**C**–**F**) = 50 µm.

On cross-sections the stomatal apparatus appears formed by the two guard cells located at the level of the epidermis (in the case of the lower one) or slightly above it (in the case of the upper one) (Figure 8D,E). The guard cells have obvious upper cristae and reduced lower ones; the ventral and dorsal walls are thicker. The stomatal apparatus is of the anomocytic type. The cauline leaf has a structure similar to the basal one (Figure 11). The thickness of the lamina is reduced; vascular bundles have a similar structure. Mesophyll appears homogeneous, undifferentiated into spongy and palisade parenchyma. All assimilatory cells are rounded, with aeriferous spaces between them. The calcium oxalate crystals are large. The leaf blade is amphistomatic.

### 2.4. Flower Structure (Figure 12 and Figure 13)

**Figure 12:**
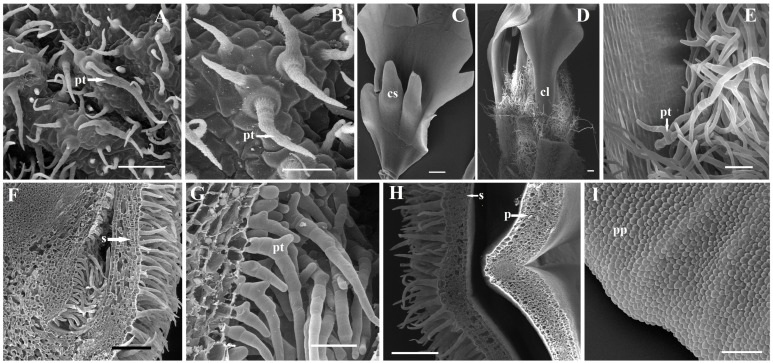
Calix and corolla (SEM micrographs): (**A**,**B**)—abaxial epidermis of the calyx, (**C**)—adaxial surface of the petal, (**D**,**E**)—abaxial surface of the petal, (**F**,**G**)—longitudinal section through sepal and petal, (**H**)—cross-section through sepal and petal, (**H**) = 200 µm, (**I**)—petal apical zone—upper epidermis: cl—claw, cs—coronal scales, p—petal, pp—petal papillae, pt—trichome, s—sepal. Scale bar: (**C**) = 500 µm, (**A**,**D**,**F**) = 200 µm, (**B**,**E**) = 100 µm, (**I**) = 100 µm, (**G**) = 50 µm.

The globular calyx is formed by five confluent sepals. The lower (external) epidermis of the sepals is covered by uniseriate multicellular non-glandular trichomes (Figure 12A,B). As a rule, these are separated from each other, but in some areas, especially in the lower part of the sepals, the bases of the trichomes are partially confluent, creating the appearance of bifurcated or trifurcated hairs.

The petals are bilobed, with an elongated claw at the base and have two coronal scales (Figure 12C). These have an average of 1.7 mm, with 1 mm of free part and a width of 0.6 mm. The petals have numerous multicellular non-glandular trichomes at the base, some uniseriate, others multi-branched, different from those found on the leaf margin or on the sepals (Figure 12D,E). The same types of trichomes, with the predominance of the uniseriate ones, are also found on the base of the staminal filaments. Outside the basal areas of the petals and staminal filaments, trichomes are absent; moreover, trichome initiation can be observed in the lower fourth of these floral parts (Figure 12F,G,H).

**Figure 13:**
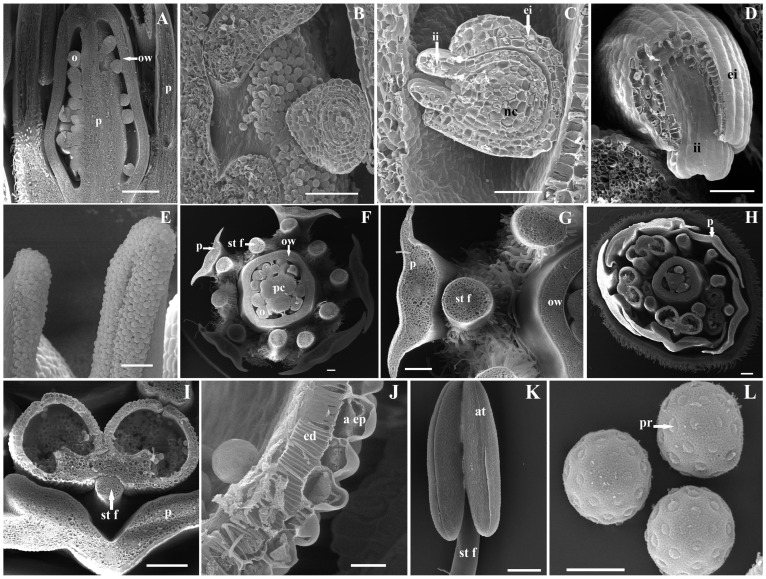
Gynoecium and androecium (SEM micrographs): (**A**,**B**)—Longitudinal section through ovary, (**C**,**D**)—longitudinal section through ovule, (**E**) stigma, (**F**,**G**)—cross-section through 1/3 basal part of the flower, (**H**,**I**)—cross-section through middle part of the flower, (**J**)—cross-section through anther wall, (**K**) at—anther—ventral part, (**L**)—pollen grains: a ep—anther epidermis, ed—endothecium, ei—external integument, ii—internal integument, nc—nucellus, o—ovule, ow—ovary wall, p—petal, pc—placenta, pr—pores, st f—staminal filament. Scale bar: (**A**,**K**) = 500 µm, (**F**,**G**–**I**) = 200 µm, (**B**,**E**) =100 µm, (**C**,**D**) = 50 µm, (**J**,**L**) = 20 µm.

The ovary is elongated, trilocular at the base and unilocular at the apex, with central, columnar placentation (Figure 13A). The external part of the placenta (in the ovarian cavity) is partially covered with papillae (Figure 13B). Numerous anatropous to campylotropous, bitegmic, crassinucellate ovules were observed in each ovarian lodge. The micropyle is long, delimited in the terminal part by the internal integument (Figure 13C,D). The stigma is trifid, covered by fully developed papillae before anthesis (Figure 13E).

The stamens are 10 in number, arranged on two whorls of five each (Figure 13F,G,H). Five stamens are in the middle of the sepals (antesepalous) and five of the petals (antepetalous). The latter have a shorter filament in the early stages of flower development and reach equal size before anthesis. The antipetal stamens have a base confluent with the petal next to which they form (Figure 13G); also, all stamens and the base of the petals are confluent at the lower part. A well-developed nectariferous disk is found in this region. At the base of the filaments there are numerous non-glandular trichomes similar to those on the base of the petals (Figure 13F,G). After the first basal quarter of the filament, they are missing. The endothecium (mechanical layer) is located under the epidermis and is made up of slightly flattened cells, with lignified thickenings arranged radially (Figure 13J). The stamens are slightly exserted at anthesis, fertile, dorsifixed, with longitudinal dehiscence (Figure 13I,K).

The pollen grains are spherical and pantoporated. Their average diameter is 300–320 µm. The pores (42–46/pollen grain) have a diameter of 37–42 µm; the ornamentation of the grains is microperforated (Figure 13L).

## 3. Discussion

The adaptation strategies of plant species in high areas are not always the same; there is no single way to solve the problems posed by the unfriendly environment—an “archetype alpine plant” [25]. To survive in these extreme climates, plants adopt different strategies that help them cross the most unfavorable periods of the year [25,26,27,28].

*S. zawadskii* presents specific morphological characteristics as a result of adaptation to the subalpine environment—short, compact plants, which helps minimize heat loss, conserves moisture and reduce exposure to wind stress [28]. The particularities of the living environment in which *S. zawadskii* grows have determined the stabilization of structural characters that allow it to survive and cope with altitudinal stress. However, the morphology of the species, with a rosette of dense and slightly leathery basal leaves, determines the appearance of structural features less specific to plants from alpine areas; normally these often present reduced leaves, with increased hairiness or covered by a thick cuticle, with a sunken stomata [15], which are not found in *S. zawadskii*. Due to its general appearance, this species actually lives in an autonomous microclimate, partially independent of the general atmospheric conditions. This fact allows it to avoid certain adaptations specific to alpine plants, which would be achieved with increased energy consumption. The concept of “microclimate” in alpine areas, which decisively influences plant life and practically “decouples” them from the general climate of the area, was emphasized by Körner [29]. In practice, plant species experience different temperatures depending on their own habit on small spatial scales; these vary greatly depending on the topography (slope, exposure, snow) [30]. That is why, sometimes, their anatomical features do not differ from those of their “relatives” that grow at lower altitudes. On the contrary, due to the overheating that occurs at ground level during hot summer days, these species must also show adaptations for a hot climate, easily resisting temperatures over 50 °C [29].

Regarding the xylem, evidence from the literature indicates that vessel diameter is a critical factor—values below 30–40 μm reduce the risk of cavitation during freeze–thaw cycles [31,32]. At high altitudes, smaller vessel diameters further enhance hydraulic safety by minimizing the risk of freezing-induced embolism [33]. The efficiency of the raw sap conduction can be ensured by increasing the number of vessels rather than by increasing their diameter, especially in species that grow in extreme conditions. The diameter of the xylem vessels of *S. sawadskii* is similar to that described in other mountain species of the same genus (*S. acaulis*, *S. scouleri*, *S. rupestris S. suecica*) [34]; the reduced diameter of the xylem vessels in *S. zawadskii* ensures increased protection against cavitation that can be generated both by low temperatures that can occur until late after the start of the growing season (especially at night and in the morning) and by physiological drought frequently encountered in the subalpine area [35], considering also the ecological preference of the species for calcareous rocks, with rapid drainage of water from precipitation. At the level of axial organs, especially in the root but also at the base of the stem, xylem vessels arranged in clusters of different sizes on cross-sections were observed; these form redundant networks of interconnected vessels [36]—the wider ones ensure more efficient water transport, and the narrower ones, more hydrodynamically stable, take over the conduction function in case of collapse of the former ones. In contrast, the species that grow at lower altitudes, *S. latifolia*, *S. vulgaris*, *S. dioeca*, *S. nutans*, have much wider xylem vessels, 42–50 μm in diameter, suggesting that they prioritize the efficiency of sap transport over frost protection, this threat not being present in their habitat [34].

The presence of mechanical tissues, especially sclerenchyma in large quantities, characterizes especially plants that grow under less stress conditions and is therefore found in smaller quantities in species that grow at high altitudes [37]. Studies on alpine species show that floral stems lignify when conditions allow this process, ensuring the support of reproductive structures [38]. At the level of the rhizome, sclerenchyma is absent, although it is often present at the level of this vegetative organ [39]. Although the sclerenchyma ring in the stem is a common feature in *Silene* species and caryophyllaceae in general, the degree of development is different depending on the areas where the plants grow—in species from lower altitudes the sclerenchyma is better developed (*S. marschallii*, *S. propinqua*, *S. thymifolia*) [40,41] than in *S. zawadski*, where it appears only in the median area of the stem and is formed by a reduced number of layers.

A particularity of leaf morphogenesis in alpine plants is represented by the early completion of cell division processes leading to the formation of mesophyll cells [25]. While in many plant species living at lower altitudes the mesophyll cells achieve their maximum number when the leaves have reached 30–40% of their final size, in alpine plants this stage is reached earlier. Observations on leaf development in *Geum reptans* have shown that the number of cells is established at a very early stage of ontogenesis, when the leaf represents only about 8% of its final size. After this, leaf growth to full size is predominantly determined by cell expansion and the formation of intercellular spaces [29,42]. In *S. zawadskii*, the number of mesophyll cell layers no longer increases after the leaf primordia are about 500 µm. However, the marginal leaf meristem continues to function, because the growth in surface area of the leaf primordia, as well as the number of vascular bundles within them, continues after this stage. Although epidermal cells are among the first to reach maturity, the morphogenesis of non-glandular trichomes in the marginal leaf zone begins at primordia of 2100–2400 µm. These observations may be consistent with developmental patterns reported for alpine species; however, in the absence of comparative data from low altitude relatives or congeneric species, this interpretation remains tentative and requires further verification through comparative studies of *S. zawadskii* was found to be of the anomocytic type; although for Caryophyllaceae the typical stomatal apparatus is the diacytic one, as described in some species of *Silene*, such as *S. takesimensis* [43], the anomocytic type has also been previously observed in other species of this genus, for example, *Silene alba* [44].

Oxalic acid resulting from plant metabolism is one of the strongest organic acids [45], with increased potential for toxicity for plant cells, and excess calcium ions have the same negative consequences on cell function [46]. Calcium oxalate formation serves as a high-capacity regulatory mechanism for sequestering excess apoplastic calcium in plant tissues, maintaining cellular calcium homeostasis by precipitating Ca into osmotically and physiologically inert crystal forms, particularly in taxa adapted to Ca-rich environments [47].

Although calcium oxalate crystals are found in numerous plant species at all evolutionary stages, from algae to higher plants [46,47,48], the accumulation and agglomeration of druses in some vegetative organs of *S. zawadskii* reach considerable dimensions. Our investigations have shown that these druses appear already in leaf primordia of 500–600 µm, where they reach a diameter comparable to that reached in other species of the Caryophyllaceae family only at the mature leaf stage [49]. The inconsistent distribution of these biocrystals in the vegetative organs of the plant is interesting—they are absent in the rhizome, but they are extremely numerous, large in size, and grouped in the pith at the base of the flowering stem. In the middle area they are present in moderate quantity, while in the terminal area they are completely absent. A similar situation is observed in the case of the roots of *S. zawadskii*, where the crystals are absent in young roots and are numerous and large in roots with a well-developed secondary structure, being distributed predominantly in the medullary rays and in the secondary xylem parenchyma. The absence of crystals in young organs may be related to the developmental stage of these tissues. Calcium oxalate deposition is frequently associated with tissue maturation, whereas actively growing tissues often contain few or no crystals. In young tissues Ca is used in larger quantities for cell division, cell wall formation and signaling processes [47].

Although the present study documents the occurrence and distribution of calcium oxalate crystals in *S. zawadskii*, their functional significance was not directly investigated. However, previous studies have suggested that calcium oxalate may act as an “internal light diffusion system”, protecting photosynthetic tissues and helping the plant to function efficiently under conditions of strong radiation, drought and high temperatures. The crystals have been reported to scatter and disperse radiation (UV, visible and infrared) inside the leaf, instead of it being concentrated in a single point [50]. They may also direct light to less exposed regions, and in plants adapted to extreme conditions they can contribute to photoprotection by filtering and dispersing excessive solar radiation, reducing the risk of photoinhibition and limiting internal leaf heating [51]. The role of calcium oxalate crystals from *S. zawadskii*, which perform these functions, remains to be experimentally demonstrated. The flower of *S. zawadskii* presents particularities that may favor pollination in the alpine environment. The success of this process is also highlighted by the increased viability of the seeds of this species [18]. The flowers are light in color, white, because the pollinator spectrum changes with elevation. Butterflies and beetles become less important at high altitudes. Bumblebees (Apidae) become dominant and are very important in alpine regions; they are better able to withstand low temperatures and can remain active even in areas where there is snow on the ground, beginning pollination immediately after snowmelt, at temperatures around 4 °C [9,52,53]. *S. acaulis* looks to be one of the important sources of nectar for bumblebees in the alpine areas [9]. Important Hymenoptera pollinators, like medium-sized bees, are not so abundant in the alpine regions compared with the lower lands [19], but still we can find species from Apidae, Anthophoridae, Colletidae, Halictidae, Megachilidae, Tenthredinidae, Braconidae, Ichneumonidae and Formicidae [54]. Also, flies become more important as pollinators at high altitudes, together with some butterflies and moths, few Coleoptera families and Hemiptera from Miridae [9,20,55,56,57,58,59]. Pollinator species on the genus *Silene* include species from Hymenoptera, Lepidoptera, Diptera and Coleoptera [56,60,61].

Inflorescences can represent 30% of the total shoot mass in alpine taxa compared with 13% in low elevation taxa and constitute a significant cost of flowering; in *Silene acaulis* (L.) Jacq., this cost amounts to approximately 10% of the seasonal photosynthetic carbon gain [9]. In *S. zawadskii*, the occurrence of relatively few flowers arranged in lax dichasial cymes may suggest a different reproductive allocation strategy; the ecological significance of this trait remains to be investigated. On the same plant species, the pollinator diversity and activities decrease with the increasing elevation but this can be offset by the dominant role of the bumblebees as pollinators in the alpine areas, reaching 60% of the plant’s visitors, and some adaptation of the plant, like the longer duration of stigmatic receptivity in the alpine plants compared with individuals from the foothill populations [19,21].

For the reproductive success of plants at high altitude, including pollen transfer efficacy, the flower morphology can influence pollinator behavior [10]. Flowers can be larger to increase the temperature, sometimes aided by heliotropism, grouped together, with vibrant colors to produce a color effects on the ground, becoming conspicuous from a distance to attract the insects, and having long-lived flowers to increase the period for pollination [9,22,23,24,60,62,63]. The flowers of *S. zawadskii* appear consistent with a relatively generalist pollination system, attracting a diverse range of pollinators; the androecium is well developed, with numerous relatively large anthers producing abundant pollen, while the elongated styles are covered with numerous stigmatic papillae that enhance the efficient capture and retention of pollen grains. However, this interpretation is based solely on floral morphology and requires confirmation through pollination studies.

Non-glandular trichomes found on the calyx and at the base of the petals and sepals of *S. zawadskii* may contribute to water conservation and may help to attenuate the effects of temperature extremes, as well as reduce herbivory by protecting sensitive floral structures, which are often more vulnerable to herbivore attacks [64]. These proposed functions, however, remain to be experimentally verified in *S. zawadskii*. Even though alpine regions may seem isolated, due to a relative simplicity of ecosystems and weak mutualistic interactions, both the plant species and pollinators in these areas, maybe more than everywhere in the world, face strongly anthropogenic threats such as climate change, climate variability, fragmentation of favorable habitats, habitat degradation, habitat conversion, and introduced plant species, resulting in a biodiversity decline, with protection becoming imperative [52,62,65,66,67,68]. Even small alterations of the pollination systems can consequence into major ecosystem-level impacts [69].

The present study provides new insights into the complex adaptive strategies developed by endemic alpine plants under multiple environmental constraints. The integrated anatomical and reproductive traits identified in *S. zawadskii* highlight its ecological specialization and underline the importance of conserving fragile high-altitude ecosystems in the context of ongoing environmental change.

## 4. Materials and Methods

### 4.1. Material Collection

The plant material consisting of whole plants of *S. zawadskii* was collected in June 2024 from the “Rarău-Pietrele Doamnei” Nature Reserve, Suceava County, Romania. The material was preserved in 70% alcohol. The specimen voucher is deposited at the Herbarium of the Faculty of Biology, Alexandru Ioan Cuza University of Iasi (No. 208539). In the study area the climate is temperate–continental, with average annual temperatures ranging from 3.8 °C at higher elevations to 5.9 °C at lower elevations, and annual precipitation levels between 700 and 810 mm [70].

### 4.2. Histo-Anatomical Analysis

For the histo-anatomical analyses, the plant material was preserved in 70% ethanol. Cross sections-were performed using a manual microtome and a razor blade. The sections were stained either with 0.5% acidified astra blue (3–5 min), or washed in distilled water and stained in 0.25% ethanolic basic fuchsin for a few seconds [71]. Starch was identified histochemically using Lugol’s reagent (iodine dissolved in potassium iodide) [72]. Lignin was identified histochemically using phloroglucinol and hydrochloric acid (Wiesner reaction) [73]. Photographs were taken with a 60D digital camera (Canon Inc., Tokyo, Japan), using an Olympus BX41 (Olympus Corporation, Tokyo, Japan) research microscope.

Measurements for anatomical parameters from stems at different levels—xylem vessel diameter in root and stem (*n* = 20), sclerenchyma pericycle thickness (*n* = 10), calcium oxalate crystal diameter (*n* = 10), upper and lower epidermis cell height and width (*n* = 20)—were carried out using IC Measure Imaging Source software, version 2.0.0.286. For the rest of the anatomical parameters, 5 measurements were done (average values or minimum and maximum was used) (The Imaging Source, LLC, Charlotte, NC, USA). Standard deviation (SD) was calculated using Microsoft Excel 2010.

### 4.3. Scanning Electron Microscopy (SEM)

Plant material consisting of stem and leaf fragments, buds and flowers was fixed and preserved in 70% ethanol. Sections were prepared manually under a stereomicroscope using a razor blade. The samples were subsequently dehydrated through a graded ethanol series (80%, 90% and 100%) followed by acetone treatment, then critical-point dried with CO_2_ using an EMS 850 Critical Point Dryer (Hatfield, PA, USA). The dried material was mounted on aluminum stubs with conductive carbon tape and sputter-coated with a 30 nm gold layer using an EMS 550X Sputter Coater (Hatfield, PA, USA). Observations were carried out with a Tescan Vega II SBH scanning electron microscope (TESCAN, Brno, Czech Republic) (acceleration voltage—30 kV) from the Electron Microscopy Laboratory, Faculty of Biology, “Alexandru Ioan Cuza” University of Iași.

## Figures and Tables

**Table 1:** Biometric data regarding some anatomical parameters investigated in the stem (means and standard deviation).

	Top Stem	Top–Middle Stem	Middle Stem	Base Stem
Epidermic cells high (µm)	29.90 ± 4.15	20.90 ± 2.82	44.50 ± 5.38	24.05 ± 2.65
Epidermic cells width (µm)	18.89 ± 3.56	20.60 ± 4.23	39.57 ± 7.58	20.30 ± 2.62
Xylem vessels diameter (µm)	10.25 ± 1.29	12.05 ± 1.39	15.68 ± 3.243	13.05 ± 2.22
Pericycle width (µm)	-	31.36 ± 6.13	130.44 ± 13.718	79.8 ± 13.90
Druses diameter in pith (µm)	-	31.77 ± 8.24	-	96.60 ± 17.01
Druses diameter in cortex (µm)	-	31.60 ± 2.93	55.11 ± 12.43	51.11 ± 11.77

## Data Availability

The original contributions presented in the study are included in the article, further inquiries can be directed to the corresponding author.
